# Targeting Peroxisome Proliferator-Activated Receptors Using Thiazolidinediones: Strategy for Design of Novel Antidiabetic Drugs

**DOI:** 10.1155/2017/1069718

**Published:** 2017-06-05

**Authors:** Neelaveni Thangavel, Mohammed Al Bratty, Sadique Akhtar Javed, Waquar Ahsan, Hassan A. Alhazmi

**Affiliations:** Department of Pharmaceutical Chemistry, College of Pharmacy, Jazan University, P.O. Box 114, Jazan 45 142, Saudi Arabia

## Abstract

Thiazolidinediones are a class of well-established antidiabetic drugs, also named as glitazones. Thiazolidinedione structure has been an important structural domain of research, involving design and development of new drugs for the treatment of type 2 diabetes. Extensive research on the mechanism of action and the structural requirements has revealed that the intended antidiabetic activity in type 2 diabetes is due to their agonistic effect on peroxisome proliferator-activated receptor (PPAR) belonging to the nuclear receptor super family. Glitazones have specific affinity to PPAR*γ*, one of the subtypes of PPARs. Certain compounds under development have dual PPAR*α*/*γ* agonistic activity which might be beneficial in obesity and diabetic cardiomyopathy. Interesting array of hybrid compounds of thiazolidinedione PPAR*γ* agonists exhibited therapeutic potential beyond antidiabetic activity. Pharmacology and chemistry of thiazolidinediones as PPAR*γ* agonists and the potential of newer analogues as dual agonists of PPARs and other emerging targets for the therapy of type 2 diabetes are presented. This review highlights the possible modifications of the structural components in the general frame work of thiazolidinediones with respect to their binding efficacy, potency, and selectivity which would guide the future research in design of novel thiazolidinedione derivatives for the management of type 2 diabetes.

## 1. Introduction

Diabetes Mellitus is one of the major threats to human health worldwide and it will be the seventh leading cause of death in 2030 [1]. A report by WHO is of high concern that the number of people above eighteen years of age getting affected by diabetes has rapidly increased [2]. Type 2 diabetes is the predominant form of diabetes and contributes to 90% of people with diabetes around the world. Range of drugs like sulfonylureas, biguanides, glinides, and glitazones are used for the treatment of type 2 diabetes but all of them suffer from unintended effects like hypoglycemia and obesity [3–6]. Thiazolidinediones (TZDs) or glitazones are important group of drugs which are active orally in the treatment of type 2 diabetes. TZDs bind avidly to Peroxisome proliferator-activated receptor gamma (PPAR*γ* agonists) and the activation of PPAR*γ* by these drugs influences a number of genes expressed which are involved in lipid and glucose metabolism and preadipocyte differentiation. They enhance the sensitivity to insulin (insulin sensitizers) and promote the utilization of glucose by peripheral tissues [7]. Glitazones have been reported to adversely affect the body weight [8] and can cause injury to the liver [9, 10].

In view of the above, medicinal chemists attempt to discover natural leads, modify the existing drugs, or synthesize compounds with fewer side effects, which would improve the quality of living and reduce the financial burden of type 2 diabetic patients. Understanding the structure of PPAR as the molecular target for thiazolidinediones, its functions in glucose and lipid metabolism, and nature of the binding interactions between PPAR and their agonists are indispensable for the discovery and design of new antidiabetic drugs. An in-depth knowledge about the chemistry of thiazolidinediones with respect to their topology, binding groups, and stereochemistry is necessary to construct or synthesize compounds with better binding affinity and specificity for PPAR*γ*. Whether modern techniques like molecular docking or traditional structure activity relationship studies, both require the idea about the structure of small molecules/ligands which are able to exhibit an agonistic activity on PPARs to either design or synthesize new molecules as PPAR*γ* or dual PPAR*α*/*γ* agonists. Owing to the significance of these chemical and pharmacological standpoints, this review will outline the molecular structure and functions of PPAR*α*/*β* and *γ*. In this review, the structures of the existing thiazolidinediones in relation to their PPAR*γ* or PPAR*α*/*γ* dual agonistic activities and toxicities will be discussed according to the literature. Furthermore, we have attempted to delineate the structure of thiazolidinediones that is vital for the development of next generation PPAR*γ* or PPAR*α*/*γ* agonists to be used in the treatment of type 2 diabetes.

## 2. PPARs as a Molecular Target for Antidiabetic Drugs

### 2.1. Structure and Molecular Mechanisms of PPARs

Peroxisome proliferator-activated receptors form a subfamily of the nuclear hormone receptor superfamily. They are ligand-inducible transcription factors [11] that regulate genes important in cell differentiation and various metabolic processes, like lipid and glucose homeostasis, insulin sensitivity, and inflammation [12]. These receptors can be induced by the fatty acids and their metabolites from the diet and are sensors of lipids and on activation would result in redirected metabolism [13]. PPARs are explored as potential targets for the therapies of diabetes, inflammation, atherosclerosis, and hypertension [14]. So far, three isoforms, PPAR*α*, PPAR*β*/*δ*, and PPAR*γ* have been identified which are encoded by distinct genes [15]. The structure of PPARs has been studied extensively using X-ray crystallography, molecular modeling, and solvent mapping techniques with respect to the DNA binding domain/region (DBD), ligand binding domain (LBD), and coactivator binding site, providing insight into the binding modes. PPARs were examined either as homodimers or as heterodimers with RXR-*α*, in presence or absence of the ligands, coactivator peptides in a DNA bound or unbound form. PPAR*γ* is one of the proteins whose structure and interactions are studied in depth.

**Figure 1:**
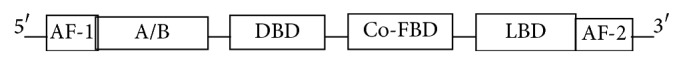
One-dimensional structure of the different binding domains of PPARs.

The 3D structure of PPARs is known to contain a DNA binding region in the N-terminal (5′) and a ligand binding region in the C-terminal (3′) of their protein structure [16]. A simple one-dimensional representation of the structure of the PPARs is given in Figure 1. The 5′, A/B domain contains a region which is independent of the ligand (AF-1), involved in the PPAR phosphorylation. The region for binding with DNA (DBD) or C domain binds to the peroxisome proliferation response element (PPRE) in the promoter region of target genes. The cofactors bind with the co-FBD/D domain. The E/F domain or ligand binding domain (LBD) binds with specific small molecules which activates the receptors leading to the targeted sequential expression of genes. AF-2 is located in the E/F domain, is ligand dependent, and binds with cofactors which aid the process of gene transcription [11].

Binding of the agonists/ligands with the ligand binding region of PPARs causes the translocation of PPARs to the nucleus and produces heterodimers with another nuclear receptor, the retinoid X receptor (RXR). The PPARs then bind with specific regions on DNA of the target genes which are named as peroxisome proliferator hormone response elements (PPREs) [17]. Nuclear receptor corepressors (NCoR), histone deacetylases (HDAC), and G-protein pathway suppressor 2 (GPS2) are examples of corepressors which prevent the binding of PPAR/RXR heterodimers with the DNA by forming high-affinity complexes with the inert PPAR/RXR heterodimers in the absence of an agonist/ligand. Activation of the PPAR takes place by binding of the agonist/ligand, leading to the displacement of the corepressor and the PPAR/RXR dimer would then bind to the PPREs in the target genes. PPRE consists of direct repeats of AGGTCA separated by a single intervening nucleotide/base pair forming the DR1. Different transcriptional coactivators/cofactors such as PPAR coactivator (PGC-1), the histone acetyltransferase p300, CREB binding protein (CBP), and steroid receptor coactivator (SRC)-1 are recruited to initiate the transcription process [18, 19]. RXRs also exist in three isoforms, forming different sets of heterodimers with PPARs, which in turn affect the promoters recognized by the target gene sequences leading to various metabolic processes. The activation process, followed by the gene transcription resulting in the changes in the expression of the regulated target genes, is similar in the all three subtypes of PPARs [20, 21].

### 2.2. Structure of DNA Binding Domain of PPARs

The A/B regions of the PPARs are poorly conserved and may act as potent transcriptional activators, get involved in protein phosphorylation, or directly interact with other receptor domains or regulatory proteins. The A/B region has not been visualized in any of the crystal structures so far studied. Moreover, it has been suggested that this region due to its high mobility does not possess significant hydrophobic residues or protected amino acid sequence to provide a meaningful binding site. PPAR*γ* ligands did not show any effect on the A/B region [22]. Two zinc-binding sites are seen in the central and highly conserved DNA binding domain, which also contains the architectural elements with the ability for sequence-specific binding to DNA [23, 24]. The structure of the DBD of the nuclear receptors was not visualized beyond the short carboxy terminal extensions (CTE) [25]. Studies on the PPAR*γ*/RXR complex have shown that, in PPAR*γ*, the carboxy terminal extension forms a significant DNA interaction and is followed by two helical segments that reach the ligand binding domain of the PPAR. The DBD has a polar arrangement and PPAR*γ* CTE is the major determinant of the polarity. The structures of PPAR*γ* show that DNA binding and the ligand binding domains are in close proximity. PPAR*γ* exhibits a selective binding with half-site AGGTCA, of the PPRE which make the DNA binding domain of the PPAR*γ* polar [22].

### 2.3. Structure of Ligand Binding Domain of PPARs

The existing isoforms of PPARs, PPAR*α*, PPAR*β*/*δ*, and PPAR*γ* bind to a variety of endogenous ligand including fatty acids and their metabolites [26]. X-ray crystallographic studies indicated that the structures of LBD in all the three isoforms are similar. The LBD of PPARs is folded into a single domain containing thirteen helices. The secondary structure is made up of three layers of antiparallel *α*-helical sandwich, H1–H12 and H2′ helices and a small four-stranded *β* sheet S1–S4. The two outer layers of the sandwich are formed by three long helices (H3, H7, and H10/H11). The middle layer of helices (H4, H5, H8, and H9) occupies the top half of the domain and is absent from the bottom half, thereby creating a very large cavity (~1400 Å^3^) for ligand binding. This large ligand binding cavity has a distinct Y shape with three arms, facilitating PPARs to bind ligand structures with branched/single chains or ligands with multiple functional groups in different conformations [27–29].

**Figure 2:**
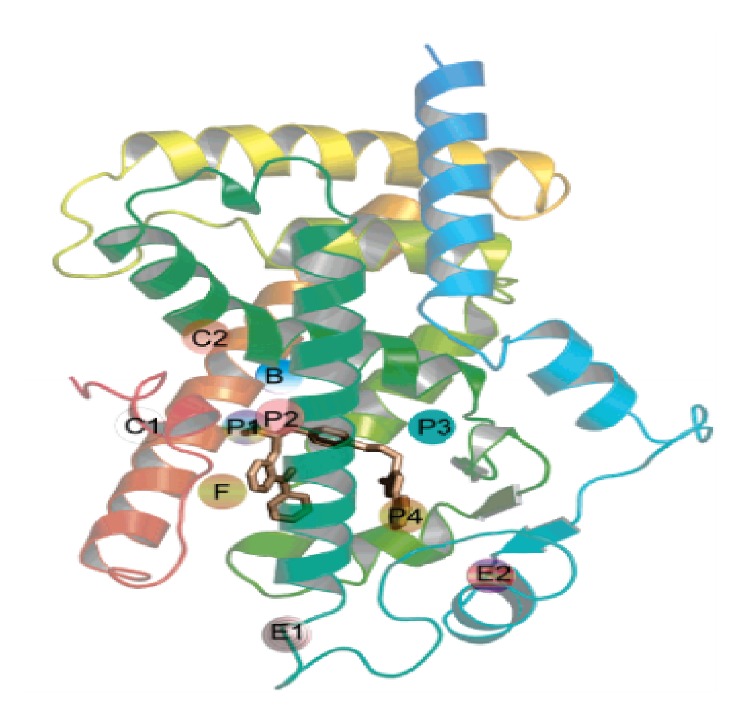
Y-shaped binding cavity with the binding sites explored using the crystal structure of the heterodimer of the human RXR*α* and PPAR*γ* ligand binding domains, respectively, bound with 9-cis retinoic acid and GI262570, a full agonist and coactivator peptides (PDB:1FM9).

Sheu and group have identified and reported ten significant binding sites in and out of the Y-shaped binding cavity of the LBD applying solvent mapping techniques on the PPAR*γ*/RXR heterodimers [30].  Figure 2 shows the binding sites of the PPAR*γ* LBD. The binding sites P1, P2, P3, and P4 are present in the Y-shaped ligand binding region. Six other binding sites are identified to be present outside the Y-shaped cavity of the LBD. Binding sites B and F are known to be on the surface of the Y-shaped binding cavity rather than inside and sites C1 and C2 are shown to be in the coactivator binding region. Binding sites E1 and E2 exist in the entrance region of the Y-shaped binding cavity.

The binding sites P1 and P2 are present on Arm I of the Y-shaped LBD and are a significant hydrophilic pocket to which all the strong agonists which interact with H12 are shown to bind. For example, the polar thiazolidinedione nucleus of the TZDs and the polar carboxyl head group of the partial agonist ragaglitazar interact with the residues in this site. Sites P3 and P4 are located at the arm II and are large hydrophobic pockets. The main ligand entrance site E2 is also hydrophobic and is present in arm III. The other binding sites which are present outside the Y-shaped cavity are involved in dimerization with RXR and interaction with coactivators.

### 2.4. Structural Variations between LBD of PPAR*α* and PPAR*γ*

**Figure 3:**
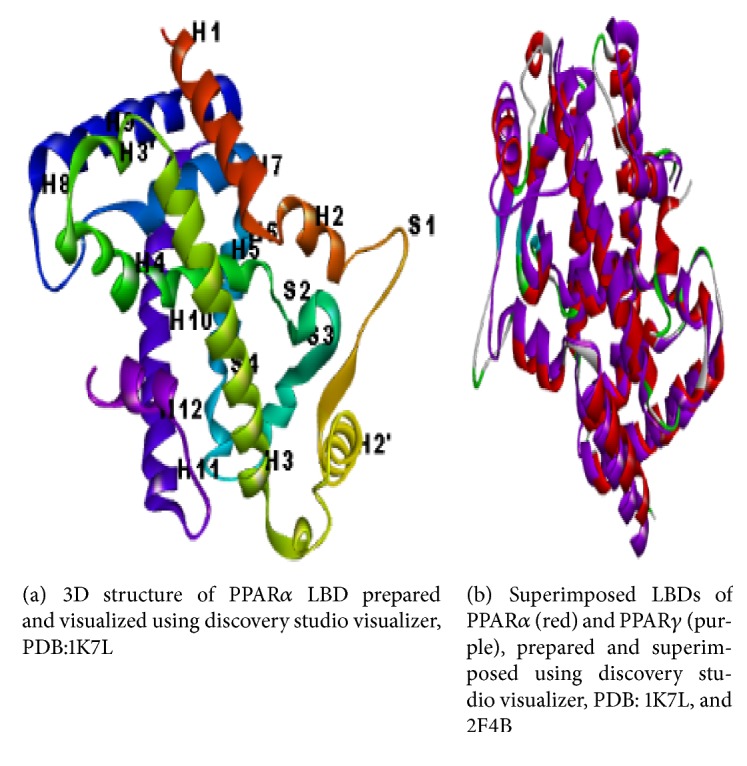


The Y-shaped binding cavity of LBD in both the isoforms is made up of 34 amino acid residues. Eighty percent of these amino acid residues and the size of the binding cavity are similar in the PPARs. The minor differences in the topology lead to ligand specificity of PPARs.  Figure 3(a) is the 3D structure of PPAR*α* LBD.  Figure 3(b) shows the similarity in the topology of PPARs.

There exist two minor changes in amino acid residues in arm I. But these changes have a marked influence on the ligand specificity since the polar head groups of the ligands interact with the binding pockets located in this arm. Arm I of PPAR*α* has Tyr314 which is replaced by His323 in PPAR*γ* and Ile354 in PPAR*α* is replaced by Phe363 in PPAR*γ*. A single change in hydrophobic arm II provides characteristic specificity for PPARs. The bulky Cys275 in arm II of PPAR*α* is replaced by Gly284 in PPAR*γ*. The most significant change in arm III, which is the ligand entrance site, is the replacement of Thr279 of PPAR*α* by Arg288 in PPAR*γ* [16].

### 2.5. Pharmacological Functions of PPAR*α*

PPAR*α* is highly expressed in tissues capable of increased fatty acid oxidation, such as liver, skeletal muscle, and heart. Activation of this receptor would result in decreased lipid levels [31]. It is also involved in glucose homeostasis and insulin resistance development [32]. Endogenous ligands like fatty acids and synthetic ligands such as fibrates are able to regulate the lipid metabolism by controlling the expression of concerned genes. Lipid metabolism in liver is largely under the control of PPAR*α*, including uptake, activation and oxidation of fatty acids, lipolysis, ketogenesis, and storage of triglycerides. PPAR*α* activation results in increased activity of lipoprotein lipase and reduces apoprotein CIII, which increases lipolysis and elimination of triglyceride-rich particles from plasma [33]. PPAR*α* act as a lipid sensor and regulate energy combustion. Activation of PPAR*α* induces genes involved in peroxisomal and mitochondrial *β* oxidation of fatty acids in hepatocytes. Cyp4A enzymes which catalyze the microsomal omega hydroxylation of fatty acids are induced by PPAR*α* ligand activation. PPAR*α* agonists decrease hepatic VLDL production and increase plasma HDL. Activation of this receptor also increases the conversion of hepatic cholesterol to HDL. Decreased hepatic gluconeogenesis and increased peripheral glucose utilization are the effects of PPAR*α* activation on glucose metabolism [34].

PPAR*α* receptor also has a significant role in the pathogenesis of fatty liver disease. Hence ligands of this receptor would be useful in the treatment of hepatic steatosis by increasing energy utilization [35].

### 2.6. PPAR*α* Agonists

Omega-3 fatty acids are the natural ligands of PPAR*α*. Docosahexaenoic acid and eicosapentaenoic acid have a carboxylic acid group which functions as a polar head group, lengthy hydrocarbon chain which functions as a linker, and a hydrophobic tail portion which are the functional groups necessary for efficient binding with PPAR*α* [36].

Fibrates are a group of synthetic PPAR*α* agonists which have been used in the treatment of dyslipidemia. The fibrate drugs decrease the apolipoprotein C-III gene expression [37]. They also increase HDL cholesterol level through an increase in apolipoprotein A-I and A-II gene expression. These drugs also decrease triglyceride level by increased fatty acid oxidation, enhance insulin sensitization, and bring down the plasma glucose levels [38]. PPAR*α* agonists have a favorable effect on the reduction of cardiovascular disease by preventing the progression of arterial lumen occlusion and PPAR*α* activation results in direct antiatherogenic effect in the artery walls [39].

**Figure 4:**
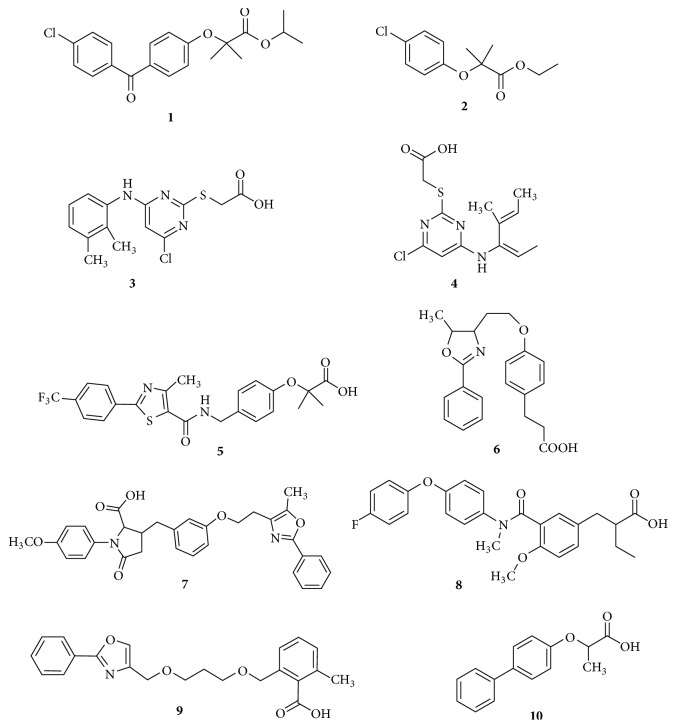
Structural features of synthetic ligands of PPAR*α*.

Synthetic PPAR*α* agonists possess a structural frame work of a carboxylic acid head linked to a hydrophobic tail through an aliphatic linker L1, central aromatic ring, and a linker L2. Structural features of synthetic ligands of PPAR*α* are shown in Figure 4. Fenofibrate** (1)** is a selective agonist of PPAR*α*. Clofibric acid, the active metabolite of clofibrate** (2),** is a dual activator of PPAR*α* and PPAR*γ*, possessing tenfold selectivity for PPAR*γ*. Pirinixic acid** (3)** and WY1** (4)** have a thioacetic acid head and function as selective PPAR*α* agonists [40]. GW735** (5)** and GW409544** (6)** contain isobutyric acid and propionic acid head, respectively. BMS631707** (7)** possess a characteristic conformationally restricted azetidinone ring linked to the carboxylic acid group, which aids the drug to occupy a new hydrophobic pocket. KRP101** (8)** has a typical topology of a PPAR*α* agonist as explained earlier [41]. Carboxylic acid head group is linked to an aromatic ring in AVE8134** (9)** structure [42]. A novel biphenyl derivative** (10)** was found to be a strong PPAR*α* agonist [43]. Till now, no TZDs have been reported as full PPAR*α* agonists.

#### 2.6.1. Binding Interactions of PPAR*α* Agonists

**Figure 5:**
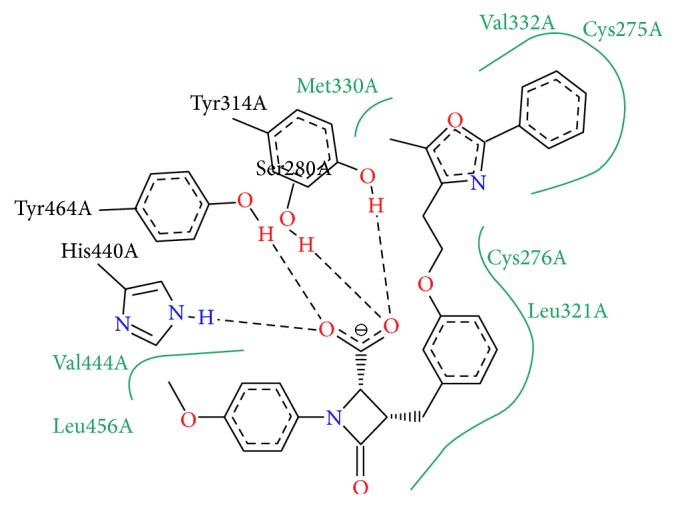
Pose view image showing the interactions of PPAR*α* agonist BMS631707, downloaded from PDB: 2REW.

Full agonists of PPAR*α* are involved in a network of hydrogen bonding interactions with helix 12.  Figure 5 shows the interaction of compound BMS631707 with PPAR*α*. The polar carboxyl head forms hydrogen bonds with His440, Tyr464, Tyr314, and Ser280. The aromatic rings occupy the hydrophobic pockets and form van der Waals contacts with Val332, Cys275, Cys276, and Leu321. The methyl groups interact by hydrophobic interactions with Met330 and Val444.

### 2.7. Pharmacological Role of PPAR*γ*

The structure and functions of PPAR*γ* have been investigated widely by the researchers and they were proved to have a significant role in regulation of lipid and glucose metabolism. PPAR*γ* is available in adipose tissue, where it regulates adipogenesis and lipid metabolism. Although this receptor is expressed from the same gene, it has different promoters and 5′ exons. This has led to the three isoforms of PPAR*γ*: PPAR*γ*1, PPAR*γ*2, and PPAR*γ*3. PPAR*γ*1 and PPAR*γ*3 are similar, while PPAR*γ*2 differs in the ligand-independent region at the N-terminus. PPAR*γ*2 has an extra 30 amino acid residues in the amino end which provides a potent transcriptional activity compared to PPAR*γ*1 [15, 44]. PPAR*γ*1 is expressed in almost all cells including adipose tissue, large intestine, hematopoietic cells, and to a lesser extent in kidney, liver, muscle, pancreas, and small intestine. PPAR*γ*2 is abundantly expressed in white and brown adipose tissue [45].

Differentiation of preadipocytes to adipocytes is a crucial phase in lipid synthesis and storage, during which PPAR*γ* is induced [46]. Adipocyte lipid storage is activated directly by the activation of genes by PPAR*γ* [47]. Activated PPAR*γ* regulates the secretion of adiponectin, leptin, and so on which have a positive effect on insulin sensitivity and also maintains the secretion of negative modulators of insulin sensitivity like resistin and tumor necrosis factor-*α* [48]. PPAR*γ* provides protection to liver and skeletal muscle and maintains their normal function by prevention of lipid overload. Besides the adipogenetic activity, PPAR*γ* modulates the genes which are important for the release, transport, and storage of fatty acids. PPAR*γ* also upregulates the expression of genes, glucose transporter type 4 (Glut4), and c-Cbl-associated protein (CAP) involved in glucose homeostasis [49]. Due to participation of PPAR*γ* in lipid and glucose metabolism, it is a target for the PPAR*γ* agonists which are used in the treatment of type 2 diabetes.

PPAR*γ* also regulates the genes related to immunity and inflammation, especially in macrophages [50, 51]. PPAR*γ* activation also results in antiatherosclerosis activity through the regulation of targets in endothelial cells and macrophages [52]. Apart from the above-mentioned activities, PPAR*γ* is also involved in the regulation of cell proliferation and apoptosis of various cancer cells including the tumors of lung, breast, colon, prostate, and bladder. PPAR*γ* activation exhibits inhibitory effect on cancer cells in vitro. Hence it may be a potential target for the development of new anticancer drugs [53, 54].

### 2.8. PPAR*γ* Agonists

Numerous researches have been carried out on this class of compounds due to their effectiveness as antidiabetic drugs. Various chemical scaffolds of natural and synthetic ligands of this group were discovered. A thorough review of the literature indicated that diverse, structurally distinct chemical compounds were able to produce PPAR*γ* activation because the ligand binding domain constitutes a large, flexible pocket able to accommodate the molecules of different size and conformations. Most of the agonists do not occupy the whole binding pocket; for example, Rosiglitazone is shown to fill up only 25% of the LBD [55]. Deep insight into the chemistry of these agonists is crucial for the rational design of novel antidiabetic drugs. Understanding the molecular mechanism of activation of PPAR*γ* by ligands with different structures and binding interactions becomes an important task to achieve a hit compound with reduced side effects and strong agonistic effect. We have arrived at a classification of PPAR*γ* agonists based on the available literature. Pharmacological and chemical classification of PPAR*γ* agonists are given in Table 1.

## 3. Implications of Glitazones in Insulin Sensitivity and Lipid and Glucose Metabolism

Glitazones are known for their antidiabetic action and belong to a group of synthetic compounds which act as agonists of PPAR*γ* and improve insulin sensitivity and reduce the blood glucose levels in patients with type 2 diabetes [56]. Activation of PPAR*γ* also enhances the sensitivity of adipocytes to insulin through modulation of the insulin signaling pathway. The beneficial effects of glitazones are due to their effect on the metabolism of lipids and glucose in liver, skeletal muscle, and adipose tissue [57].

Treatment with glitazones leads to preadipocyte differentiation in subcutaneous depots and the destruction of aged and huge insulin-resistant visceral adipocytes [58]. New and smaller adipocytes with enhanced insulin sensitivity are generated. PPAR*γ* activation by glitazones enhances the breakdown of circulating triglycerides and also increases the concentrations of adiponectin, which are low in plasma of patients with type 2 diabetes mellitus [59]. Adiponectin increases the oxidation of fatty acid, enhances sensitivity to insulin, and decreases gluconeogenesis in liver and skeletal muscle which will result in decreased levels of fatty acids, triglyceride and glucose. Besides their effect on glucose homeostasis, the glitazones inhibit the expression of insulin-resistant resistin, tumor necrosis factor -*α*, and interleukin-6 in liver and skeletal muscle [60]. Mostly, type 2 diabetes is associated with certain degree of dyslipidemia, characterized by elevated levels of triglycerides and decreased levels of HDL cholesterol. Glitazones play a beneficial role in reducing dyslipidemia [61].

Insulin is shown to increase energy storage or utilization through regulation of glucose transport into the cell, by mediation of glucose transporter glutamine 4 (GLUT4). PPAR*γ* activation by Rosiglitazone increases GLUT1 and GLUT4 expression and translocation to the cell surface, leading to the increased uptake of glucose in adipocytes and skeletal muscle, and also reduces the glucose plasma levels [62]. As a consequence of the improvement in insulin sensitivity, PPAR*γ* activation by thiazolidinediones decreases glycated hemoglobin (HbA1c) and lowers the glucose and insulin levels in patients with type 2 diabetes [63]. In addition, glitazones alleviates lipotoxicity in skeletal muscle, liver, and pancreas due to increased triglyceride synthesis leading to decreased release of free fatty acid from adipocytes. This decreases glucose synthesis by liver and increases glucose utilization in skeletal muscles, causing the hypoglycemic effects of glitazones.

### 3.1. Side Effects of Glitazone Treatment

The most common side effect of glitazones is the weight gain which is partly due to fluid retention and chiefly deposition of fat in subcutaneous tissues [64, 65]. Effects of PPAR*γ* activation by glitazones in relation to obesity and cardiovascular disease have been reported. In general, these drugs have been proved to be cardioprotective in type 2 diabetes patients [66].

Recent reports suggest that neuronal PPAR*γ* activation contributes to weight gain during treatment with Rosiglitazone and is also essential for the glitazones-mediated improvement of insulin sensitivity in liver. Glitazones would increase hunger by decreasing leptin sensitivity [67]. Interestingly, PPAR*γ* agonists have been proved to improve mood in patients with major depressive disorder accompanied by metabolic syndrome and in bipolar depression [68].

SPPAR*γ*Ms, the novel class of compounds under development, are expected to have a less impact on body weight. Metaglidasen is a SPPAR*γ*M, producing a partial PPAR*γ* agonist/antagonistic activity, has a moderate effect on adipogenesis, fatty acid uptake and synthesis. Glitazones like Balaglitazone, a partial PPAR*γ* agonist, and Netoglitazone, a dual PPAR*α*/*γ* agonist, are able to induce adipogenesis partially without affecting the body weight [69].

## 4. Structure Activity Relationship Studies on Thiazolidinediones

Relating the structure of a molecule to its pharmacological activity is an integral part of medicinal chemistry. Assessing the pharmacophores and the nature of the binding interactions becomes indispensable in enhancement of the potency and selectivity and also is important to figure out a molecule with decreased side effects. Attempts to synthesize and screen the analogues of the prototype molecule would be a suitable method for pharmacophore identification; however, it is an old, time consuming method; apart from that, it is the usual approach that provides reliable results.

**Figure 6:**
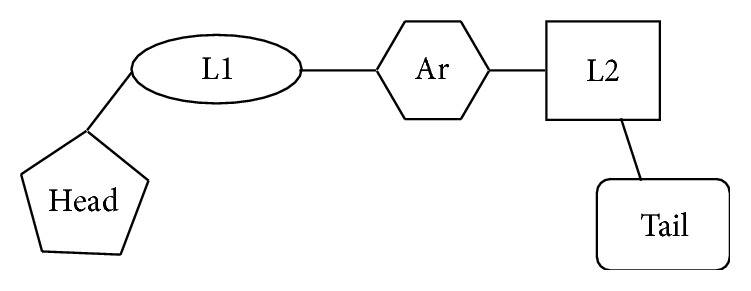
Simplified topology of a typical synthetic thiazolidinedione PPAR*γ* agonist. Geometry optimization results in a “U” shaped molecule. Head is thiazolidinedione which is acidic, polar. L1 is Linker, with not more than three carbon atoms. Ar is central aromatic/heteroaromatic ring. L2 is Linker, up to four atoms of carbon, heteroatoms. Tail is large, lipophilic groups like aromatic/heteroaromatic rings.

Sophisticated techniques employing computer software are currently available with wide range of applications in drug discovery and design. Results of such studies on synthetic PPAR*γ* agonists have suggested thiazolidinediones as a stereotype molecule for improvement. After careful observation of the literature, a simplified topology of a typical synthetic thiazolidinedione PPAR*γ* agonist is presented in Figure 6.

### 4.1. Significance of Thiazolidinedione Structure and Its Binding Interactions

**Figure 7:**
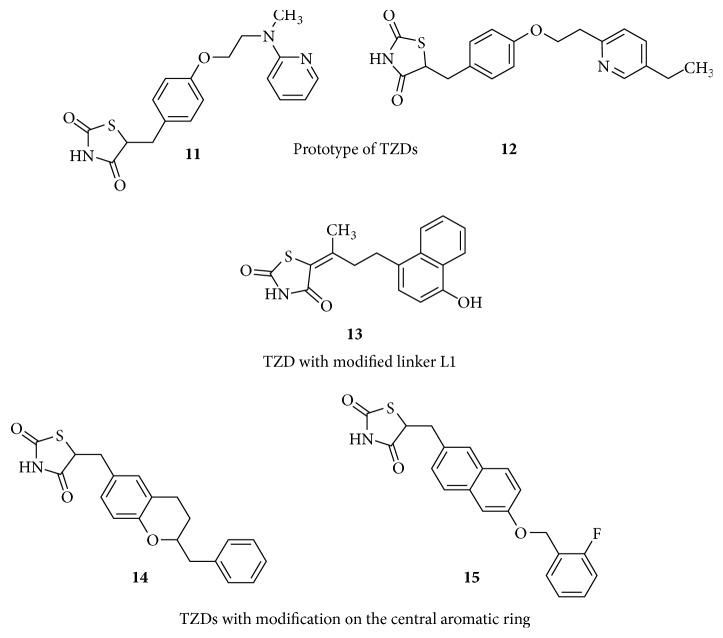
SAR of TZDs.

Glitazones contain a thiazolidine-2, 4-dione nucleus to which the linker 1 is attached at position 5. Rosiglitazone** (11)** and Pioglitazone** (12)** in Figure 7 are considered to be the lead molecules in the series of glitazone antidiabetic drugs [70]. Structural modifications on these two glitazones have been an interesting area of research in the development of novel drugs to treat type 2 diabetes. Generally, thiazolidinedione derivatives act as strong/full PPAR*γ* agonists. The binding site of PPAR*γ* is able to bind with acidic ligands through hydrogen bonds and would also interact with lipophilic ligands through hydrophobic and van der Waals interactions [71].

TZDs are designed as lipophilic acids, of which the thiazolidinedione ring behaves as the acidic head and interacts by forming hydrogen bonds with the hydrophilic binding site P_1_ present in arm I. The lipophilic tail moieties would react with the binding sites in arm II and arm III through hydrophobic and van der Waals interactions. The aliphatic linkers act as spacers, so that the acidic head and lipophilic tail are properly positioned for their efficient binding with the corresponding binding pockets of the receptor. The central phenyl ring usually provides additional hydrophobic interactions [72].

**Figure 8:**
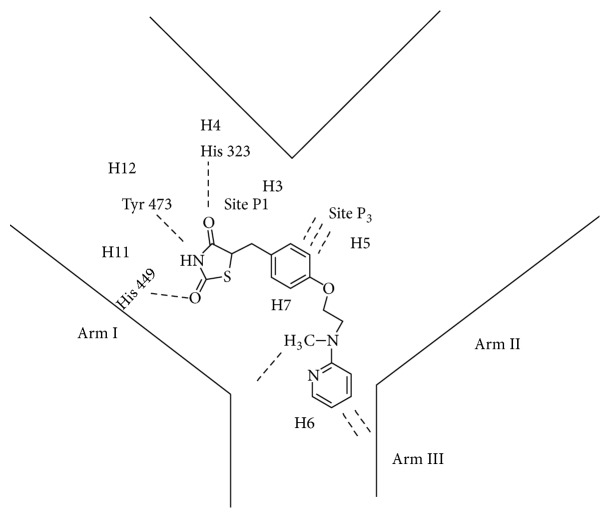
Binding interactions of Rosiglitazone with PPAR*γ* LBD.

The binding interactions of Rosiglitazone with PPAR*γ* LBD are shown in Figure 8. Rosiglitazone interacts in a U-shaped conformation with helix 12 thus stabilizing helix, which in turn recruits the cofactors [73].

Thiazolidinedione interacts by forming hydrogen bonds with His323 (H4), His449 (H11), and Tyr473 residue of helix 12 of PPAR*γ* LBD, associated with AF2 domain. It may also form hydrogen bond with Ser289 (H3) and the oxygen; nitrogen atoms of the ring function as both hydrogen bond acceptors and donors. The hydrophobic tail moiety of Rosiglitazone may also interact with helix 3, 5, 6, 7, and the *β* strand, occupying arm II and arm III of the LBD, through van der Waals and hydrophobic interactions which accounts for the efficiency of binding and potency of the molecule. The central phenyl ring is accommodated beneath helix 3 by hydrophobic interactions [74].

Regulation of transcriptional activity of PPAR*γ* depends not only on the binding of ligands and coactivators but also on its state of phosphorylation which has been explored for the invention of thiazolidinediones as partial agonists with fewer side effects, as the cyclin-dependent kinase 5 phosphorylation of Ser273 residue of PPAR*γ* has a direct influence on development of obesity. Phosphorylation of PPAR*γ* is inhibited by Rosiglitazone which is linked to the efficacy of the drug in treating type 2 diabetes [75].

A vast amount of research has been carried out in synthesizing thiazolidinedione derivatives with enhanced insulin sensitizing effect and also for their anti-inflammatory and anticancer activity [76, 77]. Modifications on thiazolidinedione ring have never been attempted, indicating the significance of this moiety. Thiazolidinedione ring is essential for PPAR*γ* agonistic activity; however, a range of lipophilic, aryl, and heteroaryl substituent could be linked to afford novel molecules with better therapeutic profile [78].

### 4.2. Molecular Modifications on L1 Linker

The glitazones already in use,** (11)** and** (12),** and those under clinical trials possess a methylene carbon atom as a linker between the thiazolidinedione ring and the central phenyl ring. Straight chain alkyl groups with maximum three carbon atoms would retain the antidiabetic activity. Linkers with more than three carbons were found to have branching in their structure. Compound** (13)** in Figure 7 has unsaturated alkyl group which may provide extra hydrophobic interactions resulting in better binding efficiency [79]. Thus, it is important to maintain the nature and length of the alkyl linker so as to space the acidic head and the lipophilic tail in their appropriate binding clefts of the PPAR*γ* LBD. Often these linkers are hydrocarbon/aliphatic chains without an electronegative atom, hence devoid of any electrostatic interaction with the polar residues of the target.

### 4.3. Molecular Modifications on Central Phenyl Ring

The central aromatic ring is significant due to its hydrophobic interactions with helix 3 of the LBD. In the classical series of glitazones, all of them consist of a phenyl group central to the chemical structure, except Englitazone and Netoglitazone which contain benzdihydropyran and naphthyl moieties, respectively. Compounds in Figure 7: Englitazone** (14)** is shown to be a strong PPAR*γ* agonist, while Netoglitazone** (15)** is able to bind with PPAR*α*/*γ* [80]. Netoglitazone due to its dual mechanism of action is expected to induce partial adipogenesis and would have a lesser effect on body weight. It is indicated in the treatment of obesity [81]. Rosiglitazone, besides its insulin sensitizing effect, activates AMPK and has a protective effect on myocardium. Netoglitazone is under clinical trials with no reported side effects so far and has a potential of being a beneficial candidate in obesity and type 2 diabetes patients with cardiovascular risk [82]. This suggests that the changes in the structure of the central aromatic ring may influence the binding mode of glitazones, resulting in distinct agonistic properties advantageous in redefining the molecules for lesser side effects.

### 4.4. Molecular Modifications on L2 Linker

The drugs, Rosiglitazone** (11)** and Pioglitazone** (12), **have different functional groups as linker, L2 between the central phenyl ring and the hydrophobic tail. The linker group in** (11)** has the maximum number of atoms among all the glitazones, with four atoms inclusive of oxygen and nitrogen atoms. It also has an N-methyl substituent on this chain. Bound conformation of Rosiglitazone with PPAR*γ* LBD shows the molecule a “U” geometry. It may be suggested that the flexibility of this linker, L2, would be vital and might assist the drug to achieve this molecular geometry. The nature of heteroatom in the chain and the length of the chain would affect the binding efficacy.

**Figure 9:**
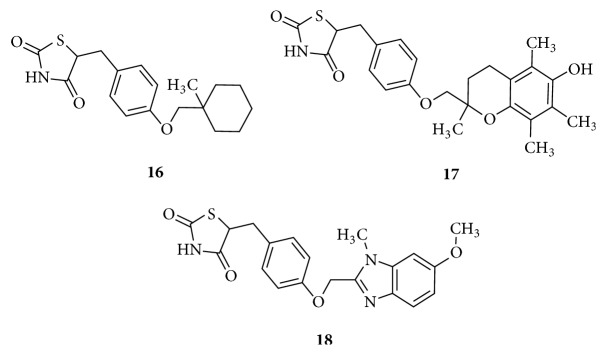
Conventional thiazolidinediones with short linker, L2.

Conventional thiazolidinediones with different linkers exhibit different binding modes and toxicity. Structures in Figure 9: Ciglitazone** (16)** was considered to be the prototype for thiazolidinediones and has a very short L2 and a different hydrophobic tail from that of Rosiglitazone but has been withdrawn from use. Similarly, other molecules such as Troglitazone** (17) **and Rivoglitazone** (18)** also possess very short L2 but were not successful drugs due to their toxicities [83].

**Figure 10:**
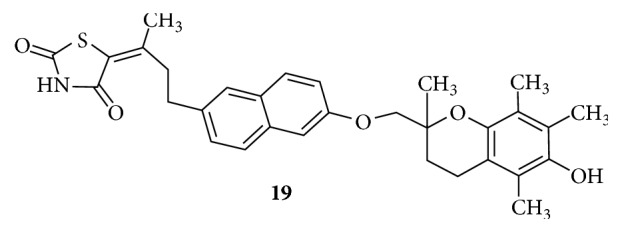
Analogue of Troglitazone.

Various analogues of Troglitazone were synthesized and tested for their effect on triglyceride and glucose levels in plasma. The analogue** (19)** represented in Figure 10, with modifications on linker L1 and the central aromatic ring, was not able to decrease the plasma triglycerides but has the ability to decrease the plasma glucose. This analogue contains an unsaturated branched L1 and a naphthyl spacer and has an intact L2 [84].

A compound called KRP-297** (20)** possesses a characteristic amido methylene group as a linker between the central aromatic ring and the hydrophobic tail. This structure also has a modified tail of trifluoromethyl phenyl ring. Interestingly this compound elicited a dual agonist function on PPAR*α*/*γ* but was found to be carcinogenic, hence withdrawn from therapeutic application [85].

**Figure 11:**
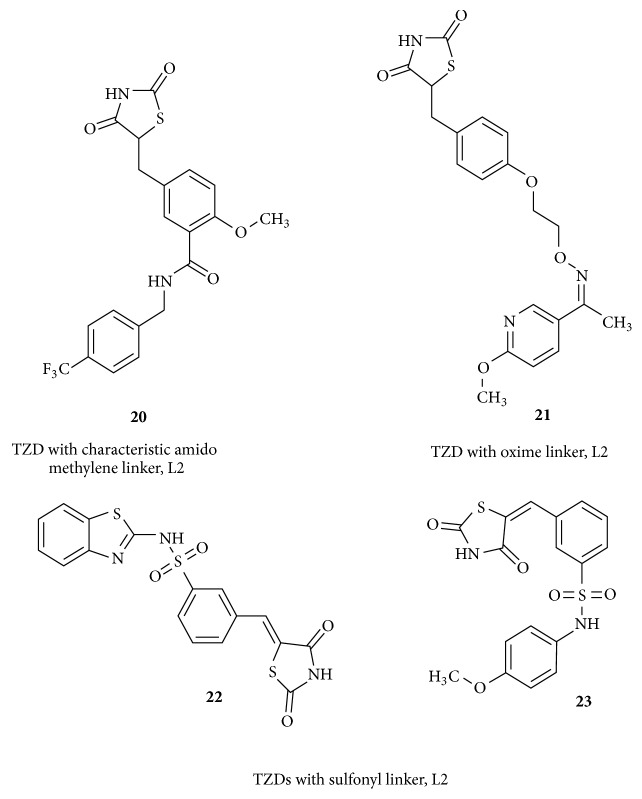
TZDs with modifications on L2.

Analogues with modifications on L2 are presented in Figure 11. The structure** (21)** having a linker, L2, with a maximum of six atoms was reported with good antihyperlipidemic, antihyperglycemic, and antiobesity properties. The presence of oxime functional group in this linker has a significant role in binding with the receptor through hydrophobic interactions [86].

A very unusual linker group like sulfonyl was used in an attempt to synthesize derivatives of thiazolidinediones as antidiabetics. Compound** (22)** bears a sulfonyl moiety in place of L2 and has a distinct highly lipophilic tail of benzothiazole, which was moderately antidiabetic. A series of simple thiazolidinediones bearing sulfonyl L2 was reported to be effective orally in type 2 diabetes. Representative molecule** (23)** belongs to this series. The significance of sulfonyl group as linker is not well understood. This group may behave as the weakest acceptor of hydrogen bond and may form a shorter hydrogen bond with polar sites at the ligand entrance in PPAR*γ* LBD. It is suggested that this group may form* syn* oriented hydrogen bonds planar with the axis of the S=O group [87].

### 4.5. Molecular Modifications on Lipophilic Tail

In a drug-receptor interaction, the binding affinity of the drug is mostly determined by the extent of hydrophobic surface which indulges in the reaction with the protein [88]. The phenomenon of desolvation of the ligand and the hydrophobic binding pockets of the protein, during the binding of the hydrophobic group, has a greater effect on the binding affinity of the ligand [89, 90]. Hence the key factor in docking the ligands with the proteins is to verify the hydration status of the unbound protein and to match the important lipophilic binding groups with the lipophilic binding site on the protein [91]. Moreover, it is observed that the binding energy is at its least when there is an optimal binding of the ligand's lipophilic group with the nonpolar binding sites of the protein [92, 93]. The size of the hydrophobic group is crucial because it is understood that the optimum binding occurs when 55% percent of the volume of the protein is filled [94].

**Figure 12:**
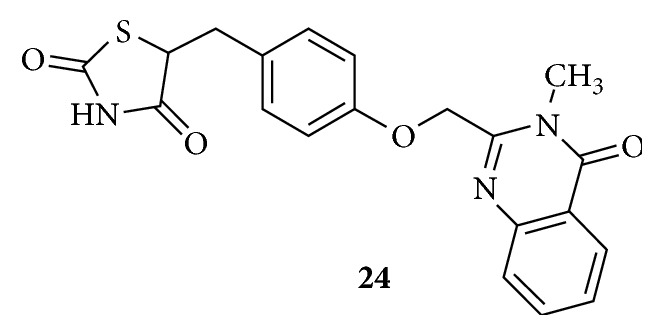
Balaglitazone, partial agonist of PPAR*γ*.

PPAR*γ* LBD has sites P3 and P4 which are large hydrophobic pockets. It is worth mentioning that Rosiglitazone** (11) **is considered to be a small molecule with a large size pyridine ring as the lipophilic tail moiety and is unable to reach P4. It is able to make hydrophobic contacts only with the binding sites of P3 and behaves as a strong PPAR*γ* agonist, whereas Balaglitazone** (24)** in Figure 12 has a bulky benzopyrimidinone moiety as the hydrophobic tail is shown to be a partial agonist of PPAR*γ*. This is well correlated with the view that partial agonists are able to occupy both the hydrophobic sites P3 and P4. Hence introducing bulky fused polynuclear or fused heterocyclic aromatic rings would alter the mechanism of action, increase the binding efficacy by increasing the volume occupied, and may also provide extra hydrophobic interactions. Balaglitazone quoted as the second generation PPAR*γ* agonist, developed by Dr. Reddy's labs of India, is under phase III clinical trial. This drug is more effective than Pioglitazone and has lesser risk of fluid retention and cardiac arrest and is also safe on bones [95].

Currently PPAR*γ* partial agonists are of interest due to their ability to produce the desirable clinical actions without usual side effects. Binding of the drug induces specific conformational variations leading to the transcriptional changes. The ultimate biological action could be fine-tuned by modulating specific coregulators, so that the antihyperlipidemic and antihyperglycemic activity is retained without producing the adverse effects of the currently used Rosiglitazone and Pioglitazone. This specific modulation of the transcriptional activity may be achieved by structural modification on the existing thiazolidinedione drugs [96, 97].

Thiazolidinedione topology is such that it can accommodate varieties of ring structures with variation in size and lipophilicity. Based on the topology and the lipophilic tail of thiazolidinediones we are providing here a classification of the analogues, in Table 2.

**Figure 13:**
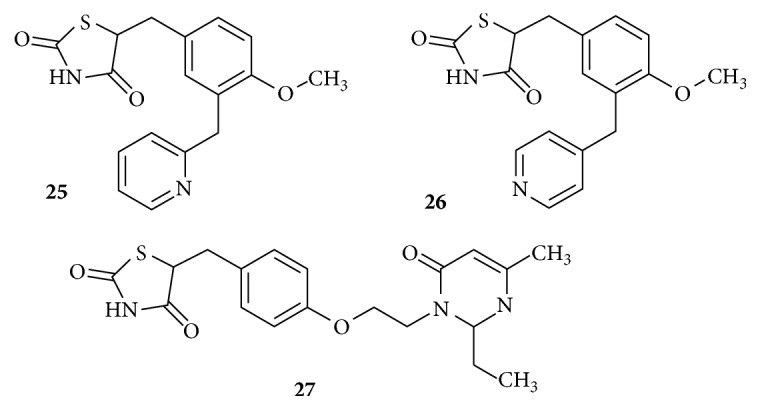
Pyridyl and Pyrimidyl analogues of TZD.

Structures of Pyridyl and Pyrimidyl analogues are shown in Figure 13. Pyridyl TZD analogues were found to increase the agonistic activity at micromolar concentrations. These derivatives possess classical linker 1 and central phenyl ring but have an amide as linker L2. Compound** (25)** was more active than compound** (26), **which might be due to the stereochemistry of the pyridine ring being favorable for its positioning in the hydrophobic pocket [98].

A Pyrimidyl TZD analogue was reported** (27)** to exhibit a better PPAR*γ* agonistic followed by transcriptional activity than Rosiglitazone and Pioglitazone. Better oral absorption and significantly less adverse reaction have been observed for this derivative [99]. Careful observation of the lipophilic tail indicates that the pyrimidine ring with its substituted alkyl groups would obey the 55% rule of the volume occupied. Hence it is expected to have a better binding affinity than the pyridyl analogues.

**Figure 14:**
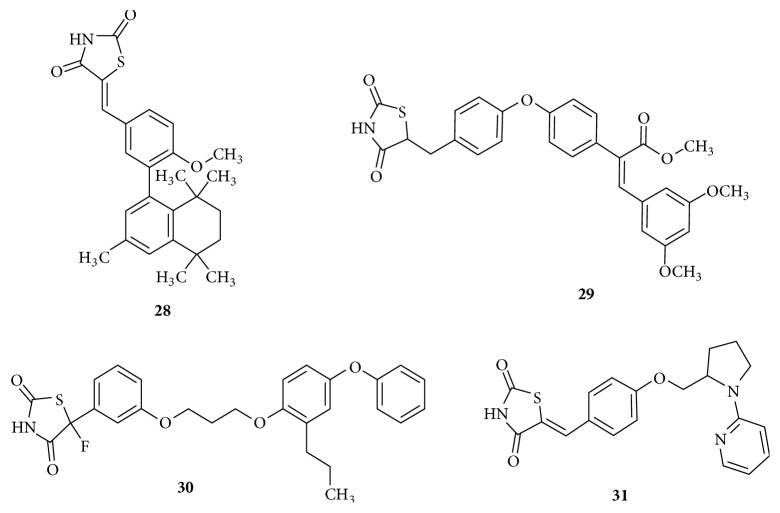
Thiazolidinedione analogues with bulky hydrophobic tail.

Analogues possessing bulky hydrophobic tail are represented in Figure 14. Tetrahydronaphthalene derivative,** (28)** was designed with an unsaturated linker L1, a central phenyl ring directly attached to the lipophilic naphthyl tail. This compound lacks L2 and elicits a moderate antidiabetic action [100]. This molecule would not be able to interact with whole of P3 and P4 due to its short linker. Styryl derivative of thiazolidinedione** (29)** produced a comparable PPAR*γ* agonistic activity with better antihyperglycemic effect [101]. Thiazolidinedione analogue** (30)** containing a lengthy L2 having five atoms and a diphenyloxy hydrophobic tail interestingly behaved as a dual agonist of PPAR*α*/*γ* with considerable insulin sensitizing effect [102]. Compound** (31)** with a pyridine ring separated from pyrrolidine ring as the hydrophobic tail group and an unsaturated L1 seems to be a potent antihyperlipidemic agent with PPAR*γ* agonistic activity better than Troglitazone [103].

**Figure 15:**
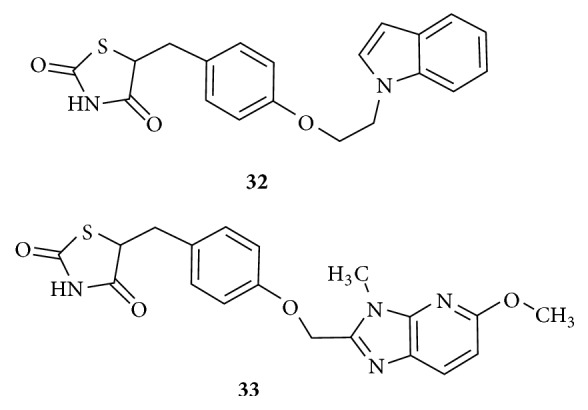
Indolyl and imidazopyridyl analogues of TZD.

Indole moiety as a lipophilic tail in analogue** (32)**, in Figure 15, with thiazolidinedione topology served as an excellent derivative with high insulin sensitizing activity compared to Rosiglitazone and antihyperlipidemic activity with enhanced HDL cholesterol levels which are beneficial compared to Troglitazone and Rosiglitazone [104]. Imidazopyridyl analogue** (33)** in Figure 15 also produced encouraging results on reduction of glucose levels with fewer side effects related to cardiovascular system compared to Rosiglitazone [105]. Indole and Imidazopyridyl moieties have behaved as best hydrophobic substituent for the tail portion for enhanced PPAR*γ* agonistic activity with decreased adverse effects. This suggests that the hydrophobic indole and imidazopyridyl moieties were able to occupy and interact with the hydrophobic binding pockets efficiently and would also have resulted in a specific modulation of transcriptional activity leading to decreased side effects.

**Figure 16:**
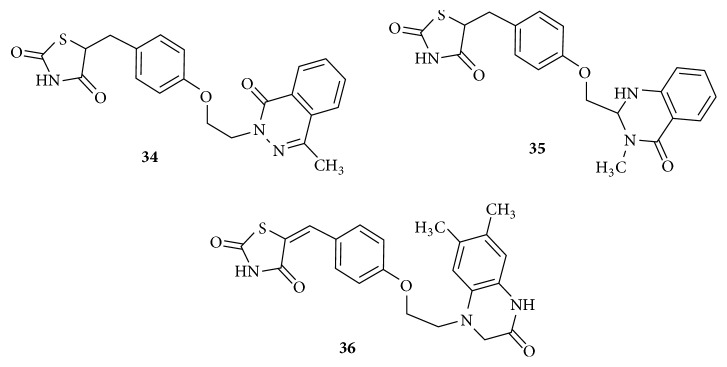
Phthalazine, quinazoline, and quinoxaline analogues of thiazolidinedione.

Among the positional isomers of pthalazinyl** (34),** Quinazolinyl** (35),** and quinoxalinyl thiazolidinediones** (36)**, as in Figure 16, the quinoxalinyl analogue was reported to be more potent than the pthalazinyl analogue. Furthermore, quinoxalinyl analogues containing activating groups like methyl at positions 6 and 7 on quinoxalinyl ring have superior glucose and triglyceride lowering activity compared to the analogues having deactivating groups such as phenyl ring. In the series of quinoxalinyl analogues compounds with fewer number of atoms in linker L2 possess significantly higher degree of antihyperlipidemic activity.

A maximum of three atoms in linker L2 would provide optimum PPAR*γ* agonistic activity. Pthalazinyl and Quinazolinyl analogues were unable to produce PPAR*γ* agonistic effect equivalent to that of quinoxalinyl derivative probably due to the steric effects leading to inefficient binding within the hydrophobic pocket [106–108].

**Figure 17:**
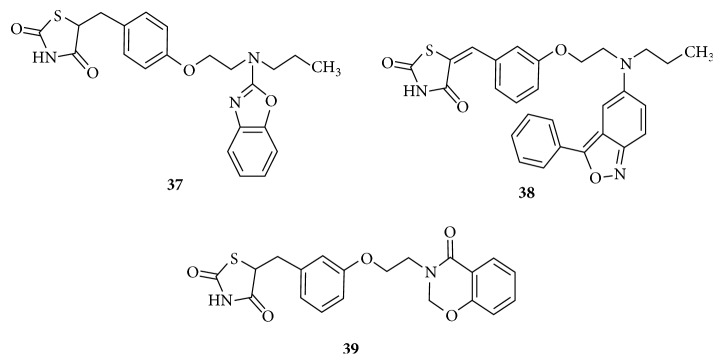
Benzoxazolyl, Benzisoxazolyl, and Benzoxazinyl analogues of thiazolidinedione.

Synthetic PPAR*γ* agonists in Figure 17 contain Benzoxazolyl** (37)** and Benzisoxazolyl** (38**) moieties as lipophilic tail of TZD were able to reduce the glucose and lipid levels. In both the series molecules with n-propyl substituent on the linker L2 exhibited the maximum activity [109, 110]. Benzoxazinyl derivative** (39)** was identified as a PPAR*α*/*γ* dual agonist indicating that ring expansion would be an advantageous strategy in designing a PPAR*α*/*γ* dual agonist.

Observation of the structures of these three series of derivatives led to the suggestion that linkers with two to three atoms are essential for optimum agonistic activity and when number of atoms in linkers were increased from three to four the molecules turn to be full PPAR*γ* agonists. Molecules with bulky moiety like benzoxazine attached through an optimum size linker of three atoms to the classical L1 and thiazolidinedione ring would result dual agonistic activity with a potential of a drug lacking side effects [111].

**Figure 18:**
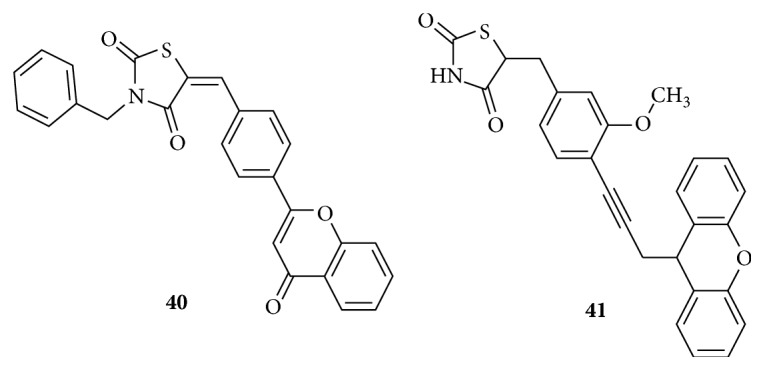
Benzpyryl and dibenzpyryl analogues of thiazolidinedione.

Benzpyryl analogue** (40)** in Figure 18, with substituent on the ring nitrogen N-3, was found to have a moderate effect on elevated glucose and lipid levels. These compounds lack the L2 linker [112]. Interesting alteration in structure of TZD was to insert a huge lipophilic ring like dibenzpyryl** (41)** with an unsaturated linker L2. It was screened for its pan PPAR*α*/*β*/*γ* agonistic activity and was proved to be effective [113].

**Figure 19:**
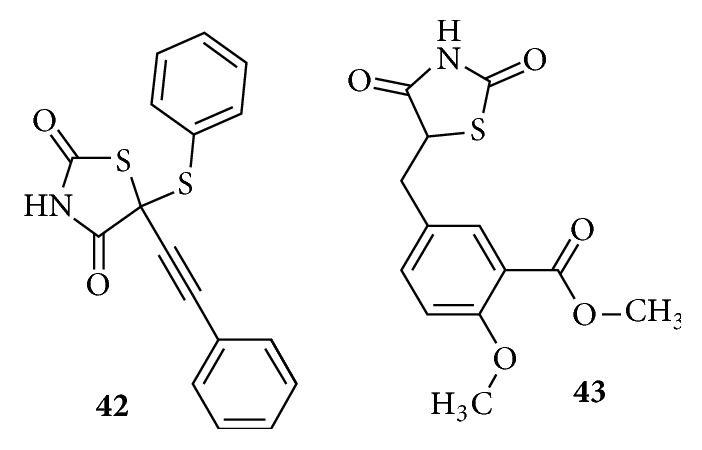
Unconventional thiazolidinediones.

Unconventional thiazolidinedione in Figure 19, possessing neither a specific linker nor a characteristic bulky lipophilic tail (**42),** is a small molecule devoid of spacer groups and shows a moderate antidiabetic activity. Simple thiazolidinediones** (43)** having medium sized groups on the central phenyl ring were synthesized as intermediates and shown to produce antihyperglycemic activity [114–117].

## 5. Hybrid Compounds of Thiazolidinedione Analogues

The concept of hybrid molecules is based on the fact that the covalent linking of two molecules with independent biological activity would result in a synergistic effect. Hybrid molecules may be composed of varieties of compounds including natural ligands, small inorganic or organic molecules, amino acids, polypeptides, and nucleic acids. These molecules are chosen through rational approach using computational methods in drug design or from compound libraries [118].

**Figure 20:**
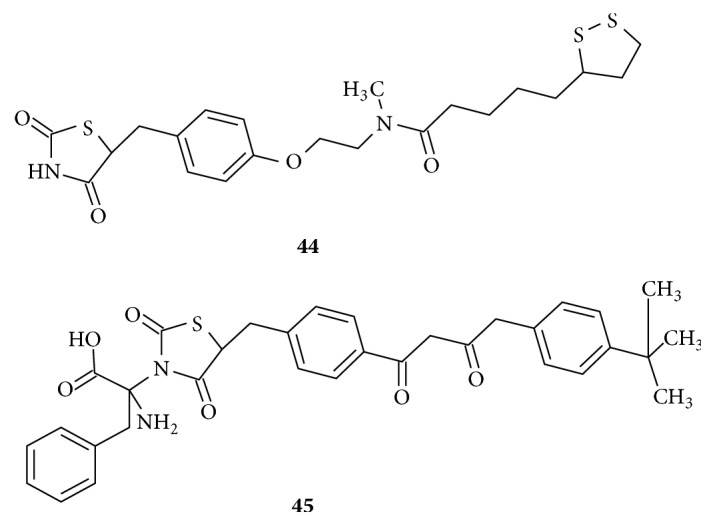
Hybrid compounds of thiazolidinediones.

Hybrid compounds are shown in Figure 20. Compound** (44)** is a hybrid of potent antioxidant *α*-lipoic acid as the lipophilic tail and the classic thiazolidinedione structure with linker L1, central phenyl ring, and L2 [119]. The significance is that potency of the hybrid molecule as PPAR*γ* agonist has been enhanced with excellent reduction in triglyceride levels. Besides their use in type 2 diabetes, hybrid molecules with various functionalities were also effective to treat obesity, vascular restenosis, and inflammatory conditions of the skin. Several other hybrid compounds have been reported with antimalignant and anti-inflammatory properties along with hypoglycemic activity [101, 120]. Compound** (45)** is a hybrid of a novel thiazolidinedione analogue with phenylalanine showing extra hydrogen bond interactions through its polar amino acid head substituted on the ring nitrogen of acidic TZD. The expected synergistic agonistic activity was achieved at nanomolar concentrations [121]. Hence hybrid compounds are currently explored as novel class of PPAR*γ* agonists for antidiabetic activity without associated risk of cardiovascular complications.

## 6. Free Fatty Acid Receptor 1 (FFAR1) as Emerging Target for Thiazolidinediones

Involvement of free fatty acids in regulation of insulin secretion by pancreatic *β*-cells has been extensively studied. The mechanism by which medium to long chain fatty acids stimulate insulin release was discovered recently that these acids stimulate G-protein coupled receptor 40 (GPR 40) otherwise termed as FFAR1. Stimulation of FFAR1 by fatty acids will enhance glucose dependent secretion of insulin from pancreas by affecting several signaling pathways inclusive of protein kinase C [122–124]. The suggested pharmacophore for activation of these receptors is presence of saturated or unsaturated carbon chain with a minimum of ten atoms and a potentially free carbonyl group [125]. FFAR1 has gained significance in recent days because they have been proved to be activated by antidiabetic thiazolidinediones [126]. Very few researches have been carried out on the molecular mechanisms of thiazolidinediones as FFAR1 agonists. Hence there exists a vast scope for design of thiazolidinediones as FFAR1 agonists/antagonists implicated in the treatment of type 2 diabetes.

**Figure 21:**
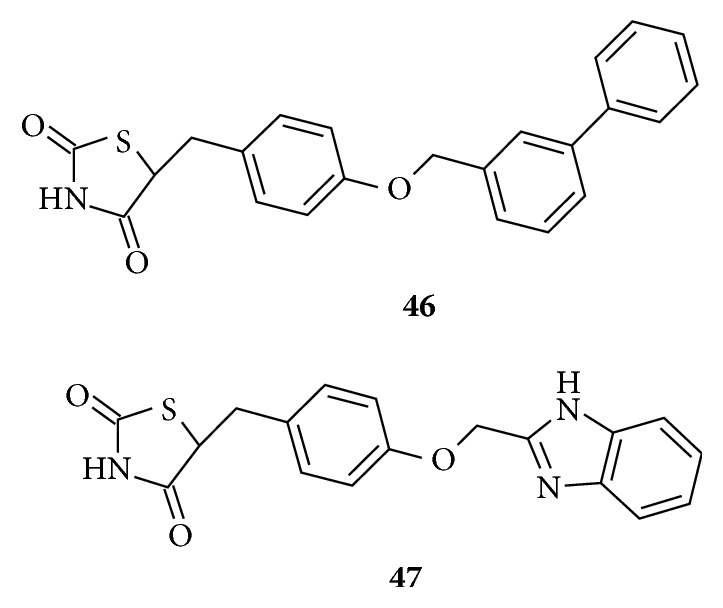
Thiazolidinedione dual PPAR*γ* and FFAR1 agonists.

New thiazolidinedione derivatives** (46 and 47)** presented in Figure 21 were found to produce dual PPAR*γ* and FFAR1 agonistic activity at micromolar concentrations with insulin sensitizing effects and enhanced insulin secretion from pancreas. These derivatives possess the classical topology of the synthetic agonists and the lipophilic tail consists of bulky groups like biphenyl and benzimidazole [127].

A study revealed that activation of FFAR1 by Rosiglitazone results in an enhancement of PPAR*γ* modulated transcription of genes [128]. Thus, PPAR*γ* and FFAR1 agonists play a pivotal role in glucose homeostasis by regulating the signal transduction of these integral dual receptors. The discovery of the concept of dual integral PPAR*γ* and FFAR1 receptor has unveiled novel methods for rational design of drugs for therapy of type 2 diabetes.

## 7. Conclusion

Structure activity relationship studies have resulted in a large pool of analogues, indicating that thiazolidinediones can accommodate varieties of linkers and lipophilic tails leading to changes in their pharmacodynamic properties, enhanced potency, selectivity, and decreased toxicity. In whole thiazolidinediones possess a large structure which enables them to bind to the polar as well as the big hydrophobic binding pockets of the ligand binding domain of PPAR. The linker (L1) between the central phenyl ring and the thiazolidinedione is at its best if it has one carbon atom. Substitution of heteroatom in this linker group did not produce any beneficial effects. The central aromatic ring is usually a phenyl ring, if expanded or replaced by a naphthyl ring would result in dual agonistic activity. The linker moiety (L2) between the hydrophobic tail and the central aromatic ring should have minimum of two atoms, at least one should be a heteroatom and a maximum of four atoms to produce optimum biological effect. The size of the hydrophobic group is an important factor in determining the binding efficacy.

In conclusion, thiazolidinediones with short/lengthy linkers and large lipophilic tail function as strong PPAR*γ* agonists and those with short linkers and bulky lipophilic tail may act as dual PPAR*α*/*γ* or PPAR*γ*/FFAR1 agonists. The binding modes of these agonists are different. Thiazolidinediones with dual PPAR*α*/*γ* agonist activity are less available because their structural requirements have not been clearly defined yet. Studies to substantiate the structure and molecular mechanisms of dual agonists of PPAR or PPAR/ FFAR1 are needed. This can be done by molecular modeling studies, targeting all PPAR receptor subtypes and FFAR1 receptor using the reported thiazolidinedione analogues.

## Figures and Tables

**Table 1:** Pharmacological and chemical classification of PPAR*γ* agonists.

(I) Strong/full PPAR*γ* agonists	
(1) Endogenous ligands:	9-HODE, 13-HODE, 15-deoxy-Δ^12,14^-prostaglandin J_2_, Prostaglandin PGJ_2_
(2) Natural ligands of plant origin:	
(a) Unsaturated fatty acids and hydroxy unsaturated fatty acids	
(b) Flavonoids:	Luteolin, Quercetin, Kaempferol, (-) Catechin 2-hydroxychalcone, Biochanin A, Genistein, 6-hydroxydaidzein
(c) Stilbenes:	Resveratrol, Amorphastilbol
(d) Amorfrutins:	Amorfrutin 1, Amorfrutin 2, Amorfrutin 3
(e) Polyacetylenes:	Falcarindiol
(f) Sesquiterpene lactones:	Deoxyelephantopin
(g) Diterpene quinone derivatives:	Sargaquinoic acid, Sargahydroquinoic acid
(3) Synthetic strong PPAR*γ* agonists:	
(a) Thiazolidinediones or Glitazones:	Rosiglitazone, Pioglitazone, Troglitazone^*∗*^, Ciglitazone^*∗*^ Rivoglitazone, Englitazone
(b) Non-thiazolidinediones:	
(i) L-tyrosine analogue:	Farglitazar
(ii) Sulfonamide derivative:	INT 131

(II) Selective/partial PPAR*γ* agonists	

(1) Thiazolidinediones:	Balaglitazone
(2) Benzodiazole derivative:	Telmisartan

(III) DUAL PPAR*α*/*γ* AGONISTS	

(1) Thiazolidinediones:	Netoglitazone, experimental compounds KRP 297, MK 767, DRF 2189
(2) Glitazars or non-thiazolidinediones	Muraglitazar^*∗*^, Tesaglitazar^*∗*^, Navaglitazar, Ragaglitazar^*∗*^ Aleglitazar

(IV) PPAR panagonists (agonists of PPAR*α*/*β*/*γ* subtypes)	

(1) Fibrate drug	Benzafibrate
(2) Miscellaneous, non-thiazolidinedione derivative	Chiglitazar
(3) Fibric acid derivative	Experimental compound ZBH201102
(4) Thiazole derivative	GW-677954
(5) Oxazole derivative	LY-465608

(V) SPPAR*γ*M (selective PPAR*γ* modulator) (Phenylacetic acid derivative)	Metaglidasen

(VI) NOVEL PPAR*γ*/FFAR_1_ DUAL AGONISTS	

(1) Thiazolidinediones	Experimental compounds KM-1, KM-2, KM-5

^*∗*^Compounds have been withdrawn from market or discontinued from clinical trials.

**Table 2:** Classification of TZD analogues.

(I) Conventional TZDs: which fit into the topology of synthetic PPAR*γ* agonist

(i)With large size rings as lipophilic tail
(a) Pyridyl TZDs
(b) Pyrimidyl TZDs
(ii) With bulky groups as lipophilic tail
(a) Naphthyl TZDs
(b) Styryl TZDs
(c) Diphenyloxy TZDs
(d) Pyridyl-Pyrrolidinyl TZDs
(iii) With fused polynuclear/heterocyclic lipophilic tail
(a) Indolyl TZDs
(b) Pthalazinyl TZDs
(c) Quinazolinyl TZDs
(d) Quinoxalinyl TZDs
(e) Benzpyryl (chroman) TZDs
(f) Benzoxazolyl TZDs
(g) Benzisoxazolyl TZDs
(h) Benzoxazinyl TZDs
(i) Dibenzpyryl TZDs
(j) Imidazopyridyl TZDs

(II) Unconventional TZDs: which do not fit into the topology of PPAR*γ* agonists

(a) TZD without characteristic lipophilic tail
(b) TZD without characteristic linkers
